# Research Progress on Micromachining Technologies Used to Fabricate Terahertz Micro-Metallic Rectangular Cavity Structures

**DOI:** 10.3390/mi16050518

**Published:** 2025-04-28

**Authors:** Xiaolei Bi, Xuemin Li, Bin Li, Xueli Cheng

**Affiliations:** 1 School of Mechnical Engineering, Henan Institute of Technology, Xinxiang 453003, China; 2 Xinxiang Additive Manufacturing Engineering Technology Research Center, Xinxiang 453003, China; 3 Department of Technology, Xinxiang Aviation Industry (Group) Co., Ltd., Xinxiang 453000, China

**Keywords:** DRIE, UV-LIGA, micro-milling, LTCC, 3D printing, electrochemical micromachining

## Abstract

Terahertz metal rectangular cavity structures are widely used in terahertz devices due to their performance advantages, and various microfabrication techniques have been applied to the manufacturing of their high performance. In this paper, several typical application fields of terahertz technology and the reasons for its application in these fields are elaborated in detail. Several typical terahertz devices with terahertz metal rectangular cavity structures are introduced in detail. The research progress of various micromachining techniques for manufacturing terahertz rectangular cavity structures, such as DRIE, UV-LIGA, micro-milling, LTCC, 3D printing, and electrochemical micromachining, is discussed in detail. Finally, the advantages and disadvantages of various micromachining techniques for manufacturing terahertz micro-rectangular cavity structures are discussed, and the results show that electrochemical micromachining technology and micro-nano 3D printing technology are relatively promising methods for the manufacturing of high-frequency terahertz rectangular cavity structures.

## 1. Introduction

Terahertz waves (also known as THz waves, T-rays, and far-infrared waves) generally refer to electromagnetic radiation with a frequency from 100 GHz to 10 THz and a wavelength of 3 mm to 30 μm, sometimes described as the “gap” in the electromagnetic spectrum [1]. This unique position endows terahertz waves with the advantages of microwaves, millimeter waves, and visible light waves, in addition to their own unique properties. Compared with microwave and millimeter waves, terahertz waves have a higher frequency, shorter wavelength, and wider bandwidth, and can be transmitted with a larger information capacity and higher resolution. Terahertz waves have lower photon energies than far-infrared waves, as well as stronger penetration and better adaptability than light waves in many harsh environmental conditions. Therefore, terahertz technology based on terahertz waves is expected to produce major technological breakthroughs in many scientific fields and have broad application prospects [2,3].

As an electromagnetic wave spectrum resource, the terahertz frequency band has not been fully developed and utilized [4]. It is therefore important to further study and understand this band as a means of creating novel research opportunities and technologies. In recent years, terahertz technology has been valued by many countries around the world, representing a new strategic direction in scientific and technological research.

As early as 2004, the US listed terahertz technology as one of the “ten technologies that will change the future world” [5]. Many institutions, including NASA, the US Naval Research Laboratory, the US Department of Energy, and the National Science Foundation, have carried out fundamental research into terahertz technology. Japan is known as a “THz superpower”, and numerous studies and investments on terahertz technology have been carried out [6]. In Europe, the Rutherford Appleton Laboratory, University of Leeds, and University of Cambridge in the UK, the Karlsruhe Institute of Technology in Germany, the Institute of Applied Physics of the Russian Academy of Sciences, and other scientific research institutions have actively studied terahertz technology, with many cross-country and multi-disciplinary collaborative research projects [7].

In 2005, Chinese scientists held an academic conference in Xiangshan with the theme of “New Development of Terahertz Science and Technology”. The development of China’s terahertz field was discussed and a blueprint for the development of terahertz technology in China was formulated. The government, universities, scientific research institutions, and enterprises of China have invested widely in terahertz research and development, with the state also providing support in related policy areas. During the “13th Five-Year Plan” period, the Ministry of Science and Technology launched a national key research and development program titled “Transformative Technology Key Scientific Issues”, a special project on “New Terahertz Radiation Sources for Biomedical Application Research”, and a national major scientific instrument and equipment development special project focused on “Coherent Strong Terahertz Source Scientific Instrument and Equipment Development”, among other projects. The National Natural Science Foundation of China funded the “Terahertz Science and Technology Frontier” Basic Science Center project and various National Natural Science Foundation projects. Terahertz communication is a key technology in achieving the vision of 6G. Hence, the Ministry of Industry and Information Technology of China established the IMT-2030 Promotion Group and released the first 6G white paper, clearly defining key technical directions such as terahertz communication and intelligent metasurfaces. A 2025 report released by the government of China clearly defined the goals of 6G, promoting the transformation of technology into commercial use.

As a forward-looking, cutting-edge, and strategic research field, terahertz technology and its applications have become priority fields for development around the world. The significance of terahertz technology to modern science, national defense, and the economy has been widely recognized. At present, terahertz technology is used in wireless communication, remote sensing detection, radar imaging, nondestructive testing, and biomedicine, among other fields, and breakthroughs in adjacent domains are highly likely [8,9].

The generation, transmission, reception, detection, and imaging of terahertz waves require the support of various terahertz devices [10]. With developments in science and technology, terahertz devices of different types and materials have been successively developed and applied. Among them are terahertz devices that incorporate metal or micro-rectangular cavity structures with metal surfaces [11]. This type of terahertz device can be effectively miniaturized and achieves high precision, although its manufacturing has stringent requirements in terms of dimensional accuracy, surface roughness, and other technical indicators. When terahertz technology is applied to the high-frequency band (that is, 1 THz and above), the required technical indicators become even more stringent. This imposes severe challenges on current micromachining technology, leading to precision machining and manufacturing becoming one of the frontiers in terahertz technology research [12]

In this paper, several typical terahertz metal rectangular cavity structures are introduced. Six micromachining methods for these terahertz metal rectangular cavity structures are described in detail: (1) deep reactive ion etching (DRIE), (2) UV-based lithography, electroplating, and molding (known as LIGA, an acronym for the German term Lithographie, Galvanoformung, and Abformung), (3) micro-milling processing, (4) low-temperature co-fired ceramics (LTCC), (5) micro-nano 3D printing, and (6) electrochemical micromachining. Finally, the advantages and disadvantages of these micromachining methods are reviewed, and the most suitable methods for the future manufacturing of high-frequency terahertz metal rectangular cavity structures are identified.

## 2. Description of Typical Terahertz Metallic Rectangular Cavity Structures

**Figure 1:**
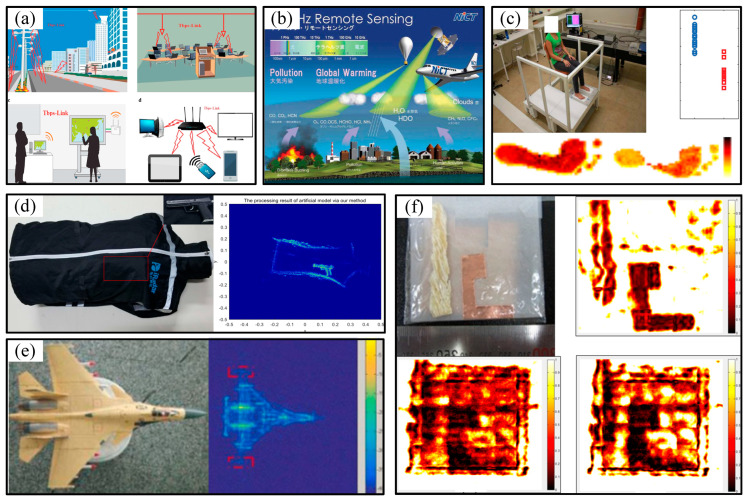
Several typical application examples of terahertz technology: (**a**) terahertz wireless communication, (**b**) terahertz remote sensing, (**c**) terahertz biomedical science, (**d**) terahertz radar imaging during safety checks, (**e**) terahertz radar imaging in the military field, and (**f**) terahertz nondestructive testing.

As the application value of terahertz technology has been gradually recognized, it has been applied to a variety of fields, as shown in Figure 1.

The rich spectral resources of the terahertz band provide possibilities for wireless transmission at rates of 10 Gbps and have the potential to reach 100 Gbps and higher. As a communication carrier, terahertz waves occupy a very wide unallocated frequency band, providing an important communication field in addition to microwave communication and optical communication [13,14]. Figure 1a illustrates the link budget for terahertz wireless data communication in the terahertz band [15]. The radiation frequency of many substances is concentrated in the terahertz band, as are the rotation and vibration spectra of most gas molecules in the atmosphere, resulting in the characteristic absorption lines of the frequency band. As such, terahertz technology has various applications in space exploration [16]. Figure 1b shows an example of terahertz remote sensing from Japan’s National Institute of Information and Communications Technology [17]. The vibration and rotation mode frequencies of biomarkers for biological lesions, such as nucleic acids, proteins, and sugars, are all in the terahertz band, and the specificity of these markers in cases of disease is reflected in the terahertz response [18,19]. Figure 1c shows an example of the application of a portable terahertz pulse imaging system, which is used by researchers at Addenbrooke’s Hospital in the UK to detect the water content of the feet of diabetic patients [20].

Terahertz radar has short wavelengths and large bandwidths; compared with microwave or millimeter wave radar, this enables a higher resolution and a better characterization of the target’s motion, fine structure, and composition [21,22]. Figure 1d shows the result of a terahertz radar imaging application involving a safety check [23]. Figure 1e shows the imaging results of a 220 GHz terahertz radar imaging system based on solid state electronics, developed by the University of Electronic Science and Technology of China [24]. Compared with X-ray detection, the single photon energy of terahertz detection is extremely low, and the risk of harm to the detected personnel and objects is minimal. The anti-interference energy of terahertz testing is stronger than that of infrared and laser nondestructive testing, allowing its application in a variety of harsh testing environments [25]. Therefore, terahertz nondestructive testing has emerged as a promising technology. Figure 1f shows an example of the application of terahertz nondestructive testing [26].

Terahertz devices are a key means of supporting the development and application of terahertz technology. Common terahertz devices include terahertz waveguides, terahertz traveling wave tubes, terahertz filters, and terahertz antennas. Terahertz waveguides are an indispensable component of terahertz wave transmission. They can be categorized, according to their structure, as solid core waveguides, hollow waveguides, porous waveguides, plate waveguides, or micro-structure waveguides, or according to their material type as metal waveguides or dielectric waveguides [27,28]. Terahertz traveling wave tubes are superior vacuum electronic devices with a wide band and high gain. Electronic vacuum devices based on a slow wave structure represent the most promising solution for generating watt-level power outputs in the terahertz frequency band while ensuring sufficiently small and economic terahertz radiation sources. The slow wave structure is the core part of terahertz traveling wave tubes. The types of slow wave structures suitable for operating in the terahertz frequency band include the folded waveguide, rectangular gate waveguide, staggered double-gate waveguide, sinusoidal waveguide, and spiral waveguide [29]. Terahertz filters allow for frequency selection and the priority extraction of characteristic signals, and can filter interference signals and noise outside of the working frequency band to effectively obtain electromagnetic spectrum information. As one of the key devices in the transceiver front-end of terahertz detection/communication systems, terahertz filters play a vital role in harmonic suppression and mirror frequency interference [30,31]. Terahertz horn antennas serve as feeders or radiators in the realization of terahertz wireless communication and detection. Horn antennas are widely used in various detector loads (corresponding to the microwave frequency band and application direction of the load product), and their performance directly affects that of the whole system. There are many kinds of feed-horns, including multi-mode horns, dielectric rod loaded horns, and corrugated horns [32,33].

**Figure 2:**
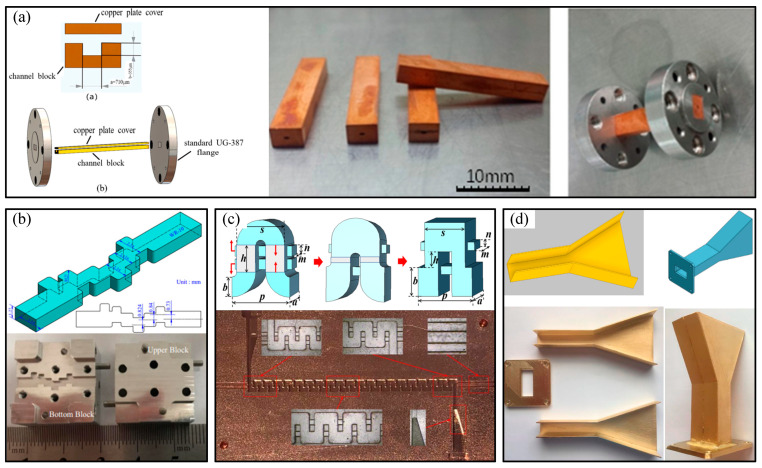
Several typical terahertz rectangular cavity structures: (**a**) 3D model and fabrication result of WR2.8 terahertz rectangular waveguide by UV-LIAG; (**b**) geometric configuration of fifth-order filter at W-band and its manufacturing results through CNC milling technology; (**c**) single-period vacuum models of the folded waveguide slow wave structure with dual-tunnel, transition structure, and the dimension parameters of the folded rectangular-grating slow wave structure, and its manufacturing detail of the FRG-SWS; (**d**) horn antenna in Autodesk Inventor program and its 3D printing results.

In recent years, the development of various advanced manufacturing technologies has led to numerous breakthroughs in terahertz devices of various types, materials, structures, and processes [34]. Terahertz devices typically have rectangular cavity structures, such as a terahertz hollow rectangular cavity, terahertz rectangular slow-wave cavity, terahertz filter rectangular cavity, or terahertz horn antenna rectangular cavity, as shown in Figure 2 [35,36,37,38]. Metal or metal-surface terahertz micro-rectangular cavity structures achieve superior performance to other materials, with low transmission loss, good flexibility, and high safety [39,40]. Therefore, the terahertz micro-metal rectangular cavity structure is widely used in terahertz devices. However, the terahertz micro-metal rectangular cavity structure required by high-frequency terahertz technology places high requirements on the machining accuracy, inner surface quality and rounded radius, and metal layer thickness and quality. Micromachining to these exacting requirements represents an urgent problem to be solved.

## 3. Manufacturing Technologies Used to Fabricate Terahertz Metallic Rectangular Cavity Structures

### 3.1. Deep Reactive Ion Etching

DRIE is a dry etching technology based on plasma bombardment of silicon surfaces to form microstructures [41,42]. Because the etched structure is not affected by crystal direction, DRIE has the advantages of high precision, a large depth-to-width ratio, and a high degree of automation. This technology is applied in the manufacturing of high-precision MEMS devices [43,44]. In the manufacturing of terahertz rectangular cavity structures, DRIE technology is first used to etch each part of the required terahertz rectangular cavity structure profile, and then surface metallization technology (e.g., surface sputtering and surface plating) is used to obtain a metal layer on the corresponding contour surface. Eutectic bonding technology is then used to combine and package the surface metallized contour structure. Finally, the desired terahertz rectangular cavity structure is formed.

**Figure 3:**
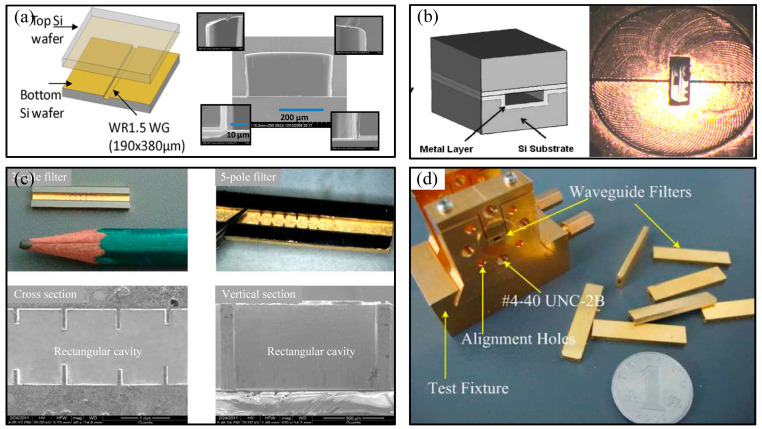
(**a**) Model and end face of the rectangular cavity structure of the WR-1.5 waveguide; (**b**) model of the rectangular cavity structure of a band pass filter in the band 385 GHz and the end face after being assembled into the flange; (**c**) cross-sectional view of a rectangular cavity of a 140 GHz bandpass filter; (**d**) photograph of a 140 GHz bandpass filter after assembly.

Using DRIE technology, researchers have achieved the fabrication of various types of terahertz micro-rectangular cavity structures. Researchers from Northrop Grumman Corporation in the United States used DRIE technology to etch a rectangular half-cavity structure with an end size of 190 μm × 380 μm on the surface of a silicon wafer, and then sputtered a gold layer on the surface, and through the gold bond with the surface sputtered gold layer of the plate. Finally, the rectangular cavity structure of a WR-1.5 waveguide operating at 500–750 GHz was prepared, as shown in Figure 3a [45]. The structure had a side-wall roughness of 75 nm, a rounded corner measurement of 92° at the side-wall position, and an average loss of 0.15 dB/mm at 600 GHz. Similarly, researchers from the Royal Institute of Technology in Sweden used the double-H plane division method with a three-wafer stack to achieve the preparation of a 220–325 GHz micro-rectangular waveguide cavity structure, which did not require special surface treatment to reduce surface roughness. The side-wall roughness was 163.13 nm, and the bottom roughness was 2.14 nm. After metallization, the thickness of the top and bottom gold layers was 0.3 μm each, the thickness of the side-wall gold layer was 1 μm, and the insertion loss measured in the whole frequency band was 0.02–0.07 dB/mm, with an average loss of 0.039 dB/mm [46].

Chinese researchers have also manufactured many terahertz micro-rectangular cavity structures using DRIE technology. Researchers from the University of Electronic Science and Technology of China used the combination of a rectangular half cavity and a flat plate with a gold layer sputtered on the surface after etching to realize the fabrication of a rectangular cavity structure applied to a 385 GHz bandpass filter, as shown in Figure 3b [47]. The section size was 0.56 mm × 0.28 mm, the thickness of the gold layer was 5 μm, the surface roughness was 0.5 μm, and the minimum insertion loss measured was 2.7 dB/mm. Researchers at the China Academy of Engineering Physics used the same method to prepare a rectangular cavity structure applied to a 140 GHz bandpass filter with a 1.651 mm × 0.825 mm cross-section. After sputtering chromium and gold on the surface, a gold layer with a thickness of approximately 6 μm was electroplated. Overall and local views of its section are shown in Figure 3c, and the final result after assembly is shown in Figure 3d [48].

### 3.2. UV-LIGA Technology

LIGA is the abbreviation of Lithographie, electroforming of Galvanoformung, and injection molding of Abformung in German. LIGA is a micro-manufacturing technology for 3D microstructures that involves lithography, electroforming, and injection molding. LIGA can produce microstructures with a depth-to-width ratio at the hundreds scale, with vertical deviations of the sidewall reaching the sub-micron level. However, this technology uses synchrotron radiation X-rays as a light source, which is expensive and complicated, thus limiting its wide application [49,50]. UV-LIGA technology is a micromanufacturing technology developed on the basis of LIGA technology. The two processes are similar, with the main difference being that UV-LIGA uses conventional ultraviolet light as the exposure light source, which greatly reduces the cost [51]. UV-LIGA technology has been successfully applied to the manufacturing of micromotors, microfluidic devices, micro-medical devices, micro-sensors, and other parts [52].

In recent years, UV-LIGA based on SU-8 photoresist has been applied in the manufacturing of terahertz micro-rectangular cavity structures. This technology usually first divides the terahertz micro-rectangular cavity structure into multiple layers; UV-LIGA based on SU-8 photoresist is then used to fabricate the corresponding microstructures on each layer, followed by surface metallization treatment. Finally, the layers are stacked and assembled using the pre-fashioned assembly holes on each layer to obtain the required terahertz micro-rectangular cavity structure.

**Figure 4:**
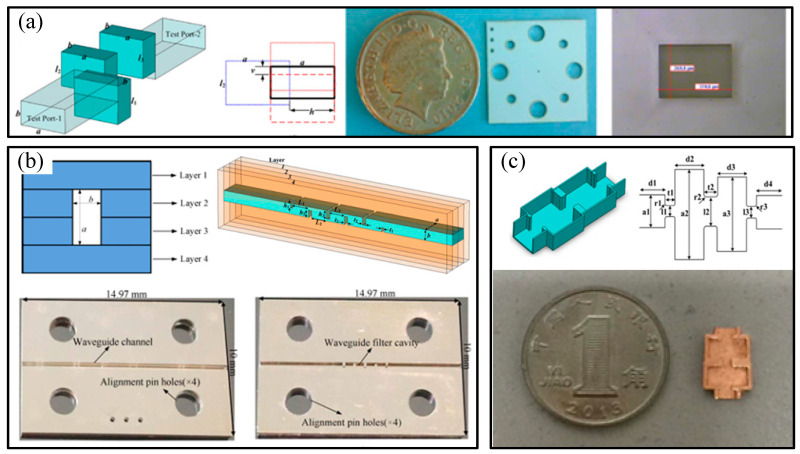
(**a**) Diagrams of a WR-1.5 band filter comprising three SU8 layers with thicknesses of 191 μm and photograph of the entire second silver-coated SU8 layer; (**b**) illustration of the fifth-order asymmetric-capacitive-iris coupled WR-3 single-band filter and photograph of one-half of a silver-coated waveguide; (**c**) schematic and topology (coupling schemes) of the filter, and the fabricated main structure of the filter.

Researchers from the University of Birmingham in the UK used the above-mentioned method to fabricate rectangular microstructures on three SU-8 photoresist layers, each with a thickness of 191 μm. Then, they silver-coated the surface to prepare a rectangular cavity structure for a third-order bandpass filter in the WR-1.5 band [53] with a side-wall roughness of 45 nm and a tolerance range of ±20 μm. The principle diagram and an example of the single-piece size measurement are shown in Figure 4a. Researchers from the University of Birmingham also used the same method to fabricate a terahertz rectangular cavity structure for a WR-3 band waveguide [54]. Each layer of photoresist had a thickness of 432 μm, and the designed dimensions of a and b were 864 μm and 432 μm, respectively. After fabricating the microstructures of each layer, a 5 nm chromium layer and a 2 μm silver layer were sputtered on the surface for surface metallization treatment. The size tolerance after assembly with pins was ±20 μm. The principle diagram and double-layer rectangular half-cavity structure after silver plating are shown in Figure 4b.

Researchers at North University of China also carried out the preparation of terahertz micro-metallic rectangular cavity structures using the UV-LIGA layer-fabrication method. A rectangular half-cavity structure for a 100 GHz bandpass filter was prepared by coating the surface of the silicon substrate with a photoresist and sputtering a silver layer, and then using three layers with thicknesses of 420 µm, 430 µm, and 420 µm successively superimposed and a sputtered silver layer. As shown in Figure 4c [55], the end face size of the cavity outlet of the structure was 2.54 mm × 1.27 mm, and the thickness of the silver layer was 1 μm. Finally, a cover plate with a sputtered silver layer was used for encapsulation.

### 3.3. Micro-Milling Technology

Micro-milling technology is a kind of ultra-precision machining technology developed based on traditional milling technology, which has the advantages of a wide selection of materials to be machined, strong 3D forming ability, high material removal rate, and good flexibility [56,57]. Objects processed by micro-milling are usually of the order of millimeters, with feature sizes usually on the micron scale. Many scientific research institutions at home and abroad have reported the application of this technology in the field of micro-machining manufacturing [58]. Micro-milling technology has been widely used in the manufacture of sub-THz vacuum electronics devices [59]. In the manufacturing of terahertz micro-rectangular cavity structures, the half cavity of the required terahertz micro-rectangular cavity structure is usually processed by micro-milling, and then the two half cavities are assembled mechanically to form the required terahertz rectangular cavity structure.

**Figure 5:**
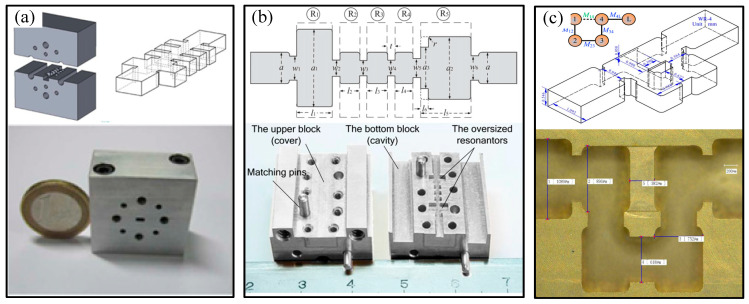
(**a**) Aspect and dimensions of the designed filter, and photograph of the fabricated filter; (**b**) initial geometry and fabricated two blocks of the filter; (**c**) coupling topology and geometric configuration of the proposed fourth-order waveguide BPF, and a top-view microphotograph of the inside of the structure.

Researchers from the University of Birmingham, UK, used a carbide cutter with a diameter of 0.2 mm to process a rectangular cavity structure with an outlet end size of 0.864 mm × 0.432 mm for a WR-3 waveguide filter on the surface of a copper alloy [60]. Researchers from Politecnica de Madrid, Spain, fabricated two rectangular half-cavity structures on the surface of an aluminum block for a four-stage asymmetric response filter in the frequency band of 100 GHz, and carried out mechanical assembly. The results are shown in Figure 5a [61], with an average manufacturing tolerance of ±10 μm and a maximum root mean square (RMS) roughness of 1 μm on the surface of the cavity machining. Researchers from Tsinghua University in China proposed a new singlet circuit based on TE301 mode, and, based on this mode, proposed a fifth-order filter with the frequency band centered at 140 GHz. The processing of the filter was realized by milling technology, with the results shown in Figure 5b [62]. Researchers from Nanjing University of Science and Technology in China proposed a 220 GHz low-loss and wideband waveguide bandpass filter with fourth-order quasi-elliptic response; the precision and roughness requirements were met by micro-milling, with the results shown in Figure 5c [63]. Researchers from Nanjing University of Information Science and Technology in China fabricated rectangular cavity structures on aluminum blocks and then electroplated a 2-μm-thick gold layer on the surface to realize the fabrication of rectangular cavity structures in a WR-3 waveguide [64].

### 3.4. LTCC Technology

**Figure 6:**
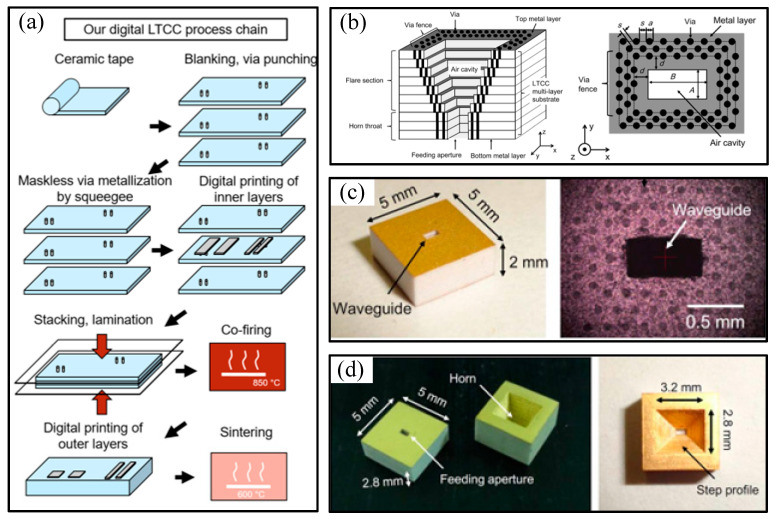
(**a**) Process chain for maskless manufacturing of high-precision LTCC; (**b**) concept of the proposed LTCC horn antenna and top view of the LTCC hollow waveguide; (**c**) photograph of the fabricated LTCC hollow waveguide; (**d**) photograph of the LTCC horn antenna prototype.

LTCC technology is a cutting-edge integrated manufacturing technique that was first proposed by the Hughes Corporation in the United States in 1982. This technology uses the tape casting process to form dense and precisely thick green ceramic tapes from ceramic slurry. Then, the required circuit patterns for each layer are produced via different processes, such as punching or laser drilling, micro-hole injection, and precise conductor paste printing. After aligning each layer, they are sintered at a low temperature of 800–900 °C to form a 3D circuit with multi-layer interconnections. It can also be made into a 3D circuit substrate with passive components inside. Subsequently, IC chips and other miniature passive components are surface-mounted on it, ultimately forming a passive/active integrated functional module or circuit [65,66,67]. The process flow is shown in Figure 6a [68]. LTCC technology can be used to design and fabricate circuits ranging from system-level to chip-scale components based on different needs. Currently, its main application areas are concentrated in three areas: high-density integration technology, high-power modules, and microwave-/millimeter-wave components [69,70].

In the field of low-frequency terahertz wave device manufacturing, LTCC technology has been employed for the packaging of related chips and devices [71]. In recent years, LTCC technology has also been utilized for the fabrication of terahertz rectangular cavity devices. Researchers from NTT Device Technology Laboratories in Japan have conducted studies in this area, and fabricated vertical rectangular waveguide cavities and rectangular corrugated horn cavities using LTCC technology, as shown in Figure 6b–d [72]. Subsequently, they integrated the two to produce a stepped corrugated horn antenna with a frequency band of 300 GHz. The rectangular waveguide cavity structure had a single-layer thickness of 0.2 mm, with a total of 20 layers, and its end face dimensions were 0.8 mm × 0.4 mm. The rectangular corrugated horn structure had a single-layer thickness of 0.2 mm, with a total of 27 layers, and its lower-end dimensions were 0.8 mm × 0.4 mm. The rectangular cavities on each substrate layer were fabricated through drilling technology, and their surface metal layer comprised a nickel/gold coating. Additionally, researchers from Device Technology Laboratories in Japan have also utilized this technology to fabricate a 300 GHz transmitter with a 3D transition structure containing a vertical hollow rectangular waveguide cavity [73].

Although LTCC technology has many advantages, such as high integration and miniaturization, because it cannot be reworked once formed, the pre-design must be strictly carried out in accordance with technical specifications to ensure parameters such as substrate size, hole and hole spacing, and through-hole conductor band coverage area. In addition, its preparation accuracy is also affected by many factors, such as the type of green tape dielectric material, lamination thickness, number of laminated layers, lamination error, drilling process, through-hole error, metal conductor band treatment method, printing error of lines and line spacing, and sintering shrinkage rate [74,75].

### 3.5. Three-Dimensional Printing Technology

Three-dimensional printing technology, also known as additive manufacturing technology, is based on computer-aided design models and 3D scanning data. It uses high-energy beam sources or other energy methods to stack and bond powders, liquids, sheets, wires, or other materials layer by layer, forming 3D structures through material superposition [76,77,78]. Based on differences in forming materials and layer-by-layer processing and stacking methods, 3D printing technology mainly includes stereolithography (SLA), which uses liquid photosensitive resin as raw material, fused deposition modeling (FDM), which uses various plastics, paraffin, and low-melting-point alloys as raw materials, and selective laser sintering (SLS) and selective laser melting (SLM), which use various metal powders as raw materials [79,80].

**Figure 7:**
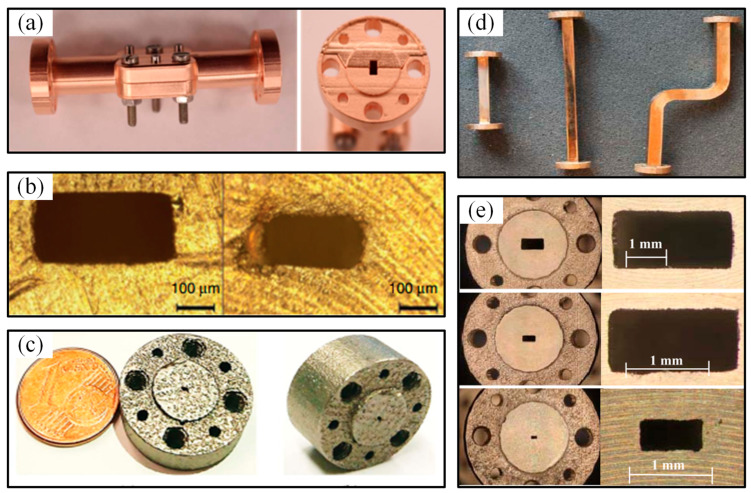
(**a**) 3D-printed metal-pipe rectangular waveguide after assembly; (**b**) micrographs of 3D-printed WM-380 and WM-250 metal-pipe rectangular waveguides; (**c**) photographs of metallic LBM SS316L 3D-printed WR3 waveguide; (**d**) photographs of SLM D-band waveguides (from left: 50 mm, 100 mm, and bend); (**e**) flange view of the SLM waveguides with E-band, D-band, and H-band.

In recent years, 3D printing technology has been applied in the manufacturing of microwave-/millimeter-wave devices, with the main manufacturing modes being “non-metallic printing + surface metallization” and direct metal 3D printing [81,82]. Among them, the “non-metallic printing + surface metallization” manufacturing mode usually uses SLA technology to print the structure of the device first, and then metallizes the inner surface, which is in contact with the electromagnetic wave. Researchers from Imperial College London used Accura Xtreme Resin as the printing material and SLA technology to manufacture a W-band (75–110 GHz) metal wire rectangular waveguide. They first printed a rectangular half-cavity structure and then metallized the surface by electroplating a 3-μm-thick nickel layer and a 27-μm-thick copper layer successively. The average surface roughness inside the cavity was measured to be 0.93 μm, and the RMS roughness was 1.16 μm. Finally, they achieved precise alignment and assembly using threaded nuts, as seen in Figure 7a [83]. Additionally, researchers from Imperial College London—by using acrylic plastic as the printing material and SLA technology—successively chemically plated a 1-μm-thick nickel layer and a 30-nm-thick gold layer, and electroplated a 1-μm-thick gold layer on the surface to successfully manufacture metal wire rectangular waveguide cavities in the frequency band greater than 500 GHz, namely WM-380 (500–750 GHz) and WM-250 (750 GHz–1.1 THz), for the first time. The results are shown in Figure 7b [84]. Transmission experiments revealed that due to the surface roughness inside the waveguide cavity, there was a significant difference between the actual transmission loss attenuation and the theoretical value. Similarly, researchers from the École Polytechnique Fédérale de Lausanne used SLA technology to prepare a terahertz linear rectangular waveguide cavity for the WR-3.4 (220–330 GHz) band. The geometric error of the printed product was ±10 μm, and it was metallized by electroplating copper on the surface followed by sputtering a 100-nm-thick gold layer [85].

The metal 3D printing mode prints the shape directly, eliminating the step of surface metallization. Researchers from the University of Duisburg-Essen in Germany used 316 L stainless steel particles with a size of 45 µm as the material and employed SLM technology to directly print a WR-3 waveguide cavity (230–320 GHz), as shown in Figure 7c [86]. Researchers from Chalmers University of Technology in Sweden studied the phase, microstructure, and surface roughness of different materials using SLM technology and directly printed metal rectangular waveguide cavity structures based on Cu-15Sn alloy for the E-band (60–90 GHz), D-band (110–170 GHz), and H-band (220–325 GHz), as shown in Figure 7d,e [87]. After cutting the printed waveguide cavity structures, a 0.8 mm × 0.8 mm range was selected to measure the average surface roughness, which was found to be 6 µm. The excessively high surface roughness was mainly due to the residual Cu-15Sn powder inside the cavity and the protrusions formed inside the cavity during printing. The cavity was then treated with a high-pressure fluid containing abrasive particles.

### 3.6. Electrochemical Micromachining Technology

Electrochemical microfabrication technology includes micro-electrolytic wire cutting technology, based on the dissolution of anode materials, and electrochemical deposition technology, based on the deposition of ions on the cathode. Micro-electrolytic wire cutting technology is an electrolytic processing technology based on the dissolution of metal anode materials, which uses metal wires with a diameter of tens of micrometers as cutting tools and ultra-short pulse generators as power sources [88]. In a specific chemical solution, the anode workpiece is cut. Through high-precision motion control, complex-shaped or high-aspect-ratio micro 3D structures/parts of different metal materials can be fabricated. Micro-electrochemical deposition is an electrochemical deposition technology based on the deposition of ions on the cathode surface. By controlling the deposition parameters, it can achieve micron- or even sub-micron-level precision processing, and realize the surface deposition and 3D formation of high-precision microstructures of pure nickel, pure copper, and other materials [89].

**Figure 8:**
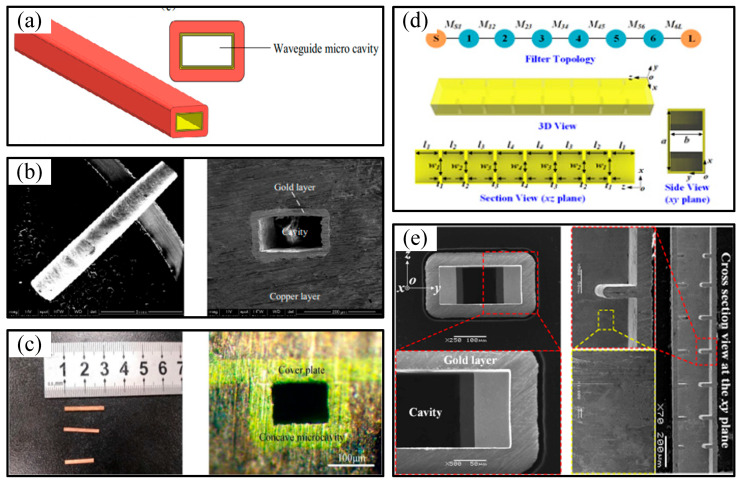
(**a**) Model of the terahertz hollow-core metal rectangular waveguide cavity; (**b**) SEM images of terahertz hollow-core metal rectangular waveguide cavity with a frequency band of 1.7 THz; (**c**) sample of combined terahertz hollow-core metal rectangular waveguide cavity; (**d**) illustration of the WR-1.0 waveguide cavity filter and its topology; (**e**) SEM images of waveguide port and internal cross section.

In recent years, researchers have achieved the manufacturing of terahertz metal rectangular waveguide cavities and terahertz filter cavities through the combined process of micro-electrolytic wire cutting technology and micro-electrochemical deposition technology. Researchers from Henan Institute of Technology improved the above-mentioned process. They first used micro-electrolytic wire cutting technology to cut a pure aluminum rectangular sacrificial core mold, and then electrochemically deposited a gold layer on the core mold surface, followed by electrochemically depositing a copper layer on the gold layer surface. Finally, the pure aluminum rectangular core mold was selectively chemically dissolved to obtain a rectangular cavity with a frequency band of 1.7 THz, a cavity end face size of 165.9 µm × 88.3 µm, and a corner radius of less than 10 μm, as shown in Figure 8a,b [90]. Researchers from Henan Institute of Technology used UV-LIGA technology to fabricate a pure nickel rectangular core mold. After sequentially electrodepositing a gold layer and a copper layer on the pure nickel surface, the rectangular half-cavity was prepared by dissolving the pure nickel rectangular core mold. Then, it was combined with a cover plate comprising a gold layer on its inner surface and a copper layer on its outer surface, and encapsulated by electrochemically depositing a copper layer to obtain a rectangular cavity with a frequency band of 1.7 THz, a cavity end face size of 81.9 × 162.7 μm, and a corner radius of less than 10 μm, as shown in Figure 8c [91]. Researchers from Southeast University fabricated high-precision rectangular waveguide cavity filter structures through electrochemical microfabrication technology. They first used micro-electrolytic wire cutting to obtain a sacrificial core mold, and then electroplated a copper layer on the core mold surface. Finally, the core mold was dissolved to obtain a high-precision waveguide cavity filter structure [92]. The proposed filter model and manufacturing results are shown in Figure 8d,e.

## 4. Conclusions

With the rapid development of terahertz technology, its future application will move towards the high-frequency band, that is, 1 THz and above. The feature size of high-frequency-band terahertz devices will further decrease, and the technical requirements for the size accuracy, surface roughness, and processing fillets of the required terahertz micro-metal rectangular cavity structure will become more stringent.

Current microfabrication technologies still have certain limitations in the manufacturing of high-frequency-band terahertz micro-metal rectangular waveguide cavities. Although DRIE technology has high manufacturing accuracy, its process is complex and the cost is high. Moreover, when the cavities are manufactured separately and then metallized and bonded, there is a problem of poor consistency in the bonding connection position. Although UV-LIGA technology has a lower cost, the method of dividing the structure into multiple pieces for separate manufacturing and then stacking and assembling them leads to problems of misalignment and poor sealing. Micro-milling technology also has problems of poor sealing after assembling the half-cavity structures, and the manufacturing of high-frequency-band terahertz rectangular cavities will be limited by the size of the micro-milling tools. LTCC technology is complex and costly, and the rectangular cavities on the single-layer ceramic sheets still must be punched or laser-drilled. The size accuracy of the single-layer rectangular cavities will be limited during the manufacturing of high-frequency-band terahertz rectangular cavities, and there will also be problems of misalignment during stacking. Three-dimensional printing technology can directly print and form the terahertz rectangular cavity as a whole, but it is limited by difficulty in internal surface metallization of micro-cavities and high surface roughness.

With the development of materials and improvement in precision, 3D printing technology represents a promising manufacturing technology for high-frequency-band terahertz rectangular cavities. Electrochemical microfabrication technology, with advantages such as material addition or removal at the ion scale, no processing stress, no heat-affected zone, and no microcracks in the melting layer, can achieve very high manufacturing accuracy and has considerable application potential in the manufacturing of high-frequency-band terahertz micro-metal rectangular waveguide cavities.
